# Prevalence of Sleep Bruxism in IBD Patients and Its Correlation to Other Dental Disorders and Quality of Life

**DOI:** 10.1155/2018/7274318

**Published:** 2018-03-12

**Authors:** C. Bucci, M. Amato, F. Zingone, M. Caggiano, P. Iovino, C. Ciacci

**Affiliations:** ^1^Gastroenterology Unit, Department of Medicine and Surgery, AOU San Giovanni di Dio e Ruggi D'Aragona, University of Salerno, Salerno, Italy; ^2^Department of Stomatology, AOU San Giovanni di Dio e Ruggi D'Aragona, University of Salerno, Salerno, Italy

## Abstract

**Background:**

Patients with inflammatory bowel diseases could experience mouth and teeth disorders and alterations in psychological mood. Vice versa, the psychological status may influence the presence of oral diseases.

**Aim:**

To evaluate in inflammatory bowel disease patients the prevalence of sleep bruxism and its correlation with the presence of oral diseases, quality of sleep, and psychological disturbances.

**Methods:**

Patients were consecutively recruited in our clinic and examined for temporomandibular disorders, dental enamel disorders, sleep bruxism, and recurrent aphthous stomatitis by two dentists. Patients also underwent Pittsburgh Sleep Quality Index and Beck Depression Inventory Scale questionnaires.

**Results:**

47 patients and 46 controls were included. Sleep bruxism and enamel wear disorders were more frequent in Crohn's disease patients when compared with ulcerative colitis patients and controls (*p* = 0.03 and *p* = 0.02, resp.). Among groups, no differences were noted for enamel hypoplasia, temporomandibular disorders, recurrent aphthous stomatitis, depression, and quality of sleep. We found a positive correlation between bruxism and temporomandibular disorders (Spearman 0.6, *p* < 0.001) and between bruxism and pathological sleep (Pittsburgh Sleep Quality Index > 5) (Spearman 0.3, *p* < 0.005).

**Conclusion:**

Bruxism and enamel wear disorders should be routinely searched in Crohn's disease patients. Moreover, the attention of healthcare givers to sleep disturbances should be addressed to all inflammatory bowel disease patients.

## 1. Introduction

Crohn's disease (CD) and ulcerative colitis (UC) are the main forms of inflammatory bowel diseases (IBD), a group of relapsing, chronic disorders of the gastrointestinal (GI) tract of unknown origin [1, 2]. Those disorders can begin at any age, with major two peaks around the 30s and 60s, a prevalence of 1.5 cases for 1000 people [3], and can proceed asymptomatically for years before a definite diagnosis is reached. Active disease is associated with an increased rate of hospitalization, surgery, and a poor quality of life [4]. IBD principally affect the gut but can also interest extra intestinal sites such as skin, eyes, liver, and joints. Patients may also experience mouth and teeth disorders. In CD, oral lesions usually are more IBD-specific (such as cobblestoning, lip and tongue fissures, mucogingivitis, and cheilitis granulomatosa) but also a-specific lesions can be observed (e.g., aphthous stomatitis) [5–8]. Conversely, in UC, only nonspecific lesions are seen and, broadly speaking, these kinds of lesions are more common than specific lesions [9].

Bruxism is a parafunctional habit defined among sleep disorders as “repetitive jaw muscle activity characterized by the clenching or grinding of teeth and/or bracing or thrusting of the mandible” [10] that can occur during the morning (awake bruxism) or night time (sleep bruxism—SB). Diagnosis of SB is more challenging than that of morning bruxism as most bruxers are unaware of their habit during night time and the majority of studies are based on self-reported questionnaires, more than on electrophysiological studies that should represent the gold standard [11]. Although nonspecific, consequences of bruxism can be tongue/cheek indentations or scalloping, abnormal tooth wear and periodontal damages, joint pathology and pain, and diffuse arthralgia [12]. Together with oral consequences, bruxism seems to be at least to some degree associated with alterations in psychological moods, such as depression or anxiety through disturbances in the central neurotransmitter system [13].

Temporomandibular disorders (TMD) are a group of chronic pathological conditions that cause pain and dysfunction in the jaw, in the muscles involved in jaw movement and associated structures. The prevalence of TMD is recognizable to be between 5% and 12%, and these data have also been confirmed in an Italian population [14]. TMD etiology is considered multifactorial, and, among others, sleep bruxism and psychological factors seem to be involved in the initiation and/or maintenance of symptoms [15].

It is known that TMD, bruxism, and enamel wear defects can be related to the psychological status of the subject and with alterations of the quality of sleep [16–18] and that mood alteration, sleep disorders, and nonspecific oral lesions are seen in patients with gastrointestinal diseases [19, 20]. However, no data exists on the prevalence of bruxism and TMD in IBD patients and on their influence on the general well-being.

The aim of the study was primarily to evaluate the prevalence of sleep bruxism in IBD patients compared to controls and secondarily its correlation to the presence of other stomatognathic system disorders (TMD, recurrent aphthous stomatitis, enamel wear defects, and enamel hypoplasia) and the quality of sleep and related psychological disturbances.

## 2. Methods

From September 2016 to March 2017, we consecutively recruited a population composed of CD and UC patients from the IBD clinic at the Gastroenterology Department of the University of Salerno, and healthy volunteers recruited among the hospital staff and friends of IBD patients. Diagnosis of IBD was made by the usual clinical, endoscopic, radiologic, and histopathologic criteria. Exclusion criteria were IBD diagnosis < 2 months, edentulous patients or older than 75 years, patients in which psychotropic drug abuse or alcoholism were known, and pregnant women. We also excluded patients who received IBD diagnosis before age 18. Clinical data were collected during the visit or from the medical records. All IBD patients had to be on stable therapy at least by 6 months at the time of the examination. Inclusion criteria for the control group were age 18, no gastroenterological diseases, and no infectious, immune-mediated, or auto-immune disorders.

After a written informed consensus, all participants were examined by the two dentists (MA and MC). The oral examination was performed by using dental mirror, explorer, and periodontal probe, and stethoscope was used for auscultation of the temporomandibular joint (TMJ) movement. All data collected were noted in a medical chart and subsequently recorded on a specific database.

During the visit, sleep bruxism was evaluated by a self-report test based on the diagnostic criteria of the American Academy of Sleep Medicine [21]. The questionnaire was composed of 4 questions for probing the possible presences of bruxism signs and symptoms during the last 6 months. In all questions, it is possible to answer only yes or no. In the first three questions, we asked the patient if he/she has ever experienced clenching or grinding the teeth during the day or the night and if he/she has noticed the presence of dental damage (enamel wear). In question number 4, we asked the patient if he/she has recurrent pain associated with TMJ dysfunction and/or with muscles involved in the masticatory activity. The test would be positive for sleep bruxism if patient's answer was yes at least one of the first three questions in addition to at least one positive answer to a symptom listed in question 4 [22].

Moreover, patients were asked about recurrent aphthous stomatitis and examined to detect temporomandibular disorders according to the Diagnostic Criteria for Temporomandibular Disorders (DC/TMD) for Clinical and Research Applications and defects of the enamel surface of the teeth (e.g., enamel wear disorder and/or enamel hypoplasia) [23]. Recurrent aphthous stomatitis (RAS) was defined as recurrent bouts of solitary or multiple recurrent shallow painful ulcers, in patients who are otherwise well [24]. Enamel wear disorder was identified by loss of mineralized tissue, due to mechanical and chemical harms [25]. In particular, we recorded the dental enamel wear defects caused by attrition because they may be the consequence of parafunctional activity like sleep bruxism. Enamel hypoplasia was defined by a reduced deposition of the enamel matrix by the ameloblasts. This alteration is clinically featured by depressions or grooves on the surface of the tooth. Depending on the severity, it can vary by a mere change of color towards white chalky or to gray and brownish, to areas of leakage of substances, up to severe cases of complete absence of enamel [26].

To analyse their psychological status and the quality of sleep, patients also underwent the Beck Depression Inventory-II (BDI-II) and the Pittsburgh Sleep Quality Index (PSQI).

The BDI-II is a most widely used self-report test for depression severity evaluation [27]. It includes 21 multiple-choice items, and it assesses both psychological and physical symptoms during the previous month. Depression total score was classified as absent (BDI-II score < 13) or mild (BDI-II score 14–19), moderate (BDI-II score 20–28), and severe depression (BDI-II score, 29–36). In this study, BDI-II was used to compare depression level in IBD patient versus control group.

The PSQI measures sleep quality during the previous month and to discriminate between good and poor sleepers [28]. The PSQI is a 19-item self-rated questionnaire plus 5 questions rated by a bed partner or roommate (only the self-rated items are used in scoring the scale). The self-administered part is made of 15 multiple-choice items investigating the frequency of sleep disturbances and subjective sleep quality and four write-in items that inquire about typical bedtime, wake-up time, sleep latency, and sleep duration. The PSQI generates seven scores that correspond to the domains listed previously. The global score may range from 0 to 21. A PSQI global score > 5 is suggestive of significant sleep disturbance [28]. The study was approved by the Ethics Committee of the University of Salerno Campania Sud.

### 2.1. Statistical Analysis

Categorical and continuous variables were expressed as frequency and mean ± standard deviation (SD), respectively. Differences in frequencies among groups were calculated using *χ*^2^ test, while differences among continuous variables were calculated using ANOVA. The univariate logistic analysis was used to evaluate the risk of sleep bruxism in CD and UC patients compared to controls. The Spearman correlation was used to test the association between the main outcome (the presence of bruxism) and the other dental disorders, the presence of depression, and pathological sleep. All tests were two-tailed with significance level set at *p* < 0.05. All analyses were performed using Stata version 12, StataCorp., College Station, TX.

## 3. Results

Fifty-three IBD patients consecutively attending the outpatient clinic at the University of Salerno were eligible to participate in the study. Of those, 2 CD and 1 UC patients refused to undergo the oral examination, and 3 CD were excluded because they were edentulous. Therefore, 47 patients (28 CD and 19 UC) were enrolled in the study. About half of CD patients were male (48.3%), mean age at test was 38.5 years (±15.4), with a mean time from diagnosis of 8.1 years (±8.5), while 57.9% of UC patients were male, mean age at test was 39.7 years (±17.4), with a mean time from diagnosis of 6 years (±6.5). Forty-seven sex- and age-matched controls were included in the study (51% were male, mean age at test 32.6 years (±9.9)). Study population characteristics are summarized in Table 1.

Sleep bruxism was more frequent in CD patients compared to UC and controls (*p* = 0.03). In particular, the risk of bruxism was 3.9 times higher in CD patients than in controls [OR 3.9 (95% CI 1.4–11.02)], while there was no statistically significant increased risk of bruxism in UC patients compared to controls [OR 1.9 (95% CI 0.6–6.53)] (Table 2). Among groups, no differences were noted for enamel hypoplasia and TMD. Enamel wear disorders were more frequent in CD patients compared to UC and controls (*p* = 0.02). Specifically, they were reported in 8 CD (28.5%), in 5 UC (26.3%) patients, and 3 controls (6.4%). In detail, one CD female patient had a grade 1 enamel hypoplasia, according to Aine classification (defects in the colour of enamel; single or multiple creams, yellow, or brown opacities with clearly defined or diffuse margins). In all other 12 IBD patients and in 3 controls, we found attrition enamel wear defects characterized by loss of mineralized tooth tissue, unrelated to bacterial demineralization action. RAS were reported in 8 CD (28.5%) and in 6 UC patients (31.6%) and in 10 controls (21.3%) (*p* = ns). Depression and quality of sleep were similar among the three groups (Table 3).

In our cohort, we found a positive correlation between bruxism and TMD (Spearman 0.6, *p* < 0.001) and between bruxism and pathological sleep (PSQI > 5) (Spearman 0.3, *p* < 0.005), but we failed to found any correlation between bruxism and RAS (*p* = 0.1), bruxism and depression (*p* = 0.8), bruxism and enamel hypoplasia (*p* = 0.5), and bruxism and enamel wear disorders (*p* = 0.2).

## 4. Discussion

Patients' outcomes are always measured with objective indicators; oral health and dental treatment make no exceptions. Healthcare of IBD patients, as for most chronic disease, implies a patient-centered approach which broadens the spectrum of interventions beyond the intestine. Even if the holistic approach to patient care emphasized the importance of treating patients as human beings instead of “body parts,” the correct approach is always to fully understand the extent of the relationship between the single sign or symptom, including mouth and teeth, and the biological, psychological, and social context.

Data from the present study showed that bruxism and enamel wear defects in CD but not UC patients show a 4-fold increase when compared to healthy controls. Also, there is a significant association between bruxism and TMD and between bruxism and pathological sleep (PSQI > 5). However, we found no correlation between bruxism and possible signs of immunological disease such as recurrent aphthous stomatitis and enamel hypoplasia.

Bruxism is an oral condition that has raised great interest in the last years for its controversial nature [29]. Some authors, in fact, consider it as a “behaviour” that may lead to oral damages more than a specific disorder itself. What is acknowledged is the existence of a link between bruxism, alterations in psychological moods, such as depression or anxiety, sleep disorders, and TMD [13, 30, 31]. The quality of life and disease activity of IBD patients is strongly influenced by their psychological status, and as a consequence, any factor that could influence their emotional stability should be considered in the global management of each patient [32]. However, we did not show any correlation between bruxism and depression in IBD, maybe because of the number of patients examined.

Our study, for the first time, indicates the need of including bruxism and TMD examination in the routine follow-up of CD patients, whereas the attention of healthcare givers to sleep disturbances should be addressed to both CD and UC patients. Close collaboration with a dentist familiar with the manifestations of oral pathology would greatly facilitate the accurate identification of oral lesions in patients presenting with CD.

Our study has the limit of the number of IBD patients enrolled. Now that the relationship has been found, a systematic review of large series of patients could shed light on the prevalence of both bruxism and TMD in IBD patients.

## Figures and Tables

**Table 1:** Study population characteristics.

	HC (*N* 47)	CD (*N* 28)	UC (*N* 19)	*p* value
Males, *N* (%)	24 (51%)	14 (48.3%)	11 (57.9%)	0.8
Age, mean (±SD)	32.6 (9.9)	38.5 (15.4)	39.7 (17.4)	0.07
Years from diagnosis, mean (±SD)	—	8.1 (8.5)	6 (6.5)	0.3

HC = healthy controls; CD = Crohn's disease patients; UC = ulcerative colitis patients; *N* = number(s).

**Table 2:** Oral examination findings in CD, UC, and healthy controls.

	HC (*N* 47)	CD (*N* 28)	UC (*N* 19)	*p* value
Sleep bruxism, yes (%)	9 (19.1%)	14 (50%)	6 (31.6%)	0.03
Enamel hypoplasia, yes (%)	0	1 (3.5%)	0	0.3
TMD, yes (%)	9 (19.1%)	7 (25%)	6 (31.6%)	0.5
Enamel wear disorders, yes (%)	3 (6.4%)	8 (28.5%)	5 (26.3%)	0.02
RAS, yes (%)	10 (21.3%)	8 (28.5%)	6 (31.6%)	0.6

HC = healthy controls; CD = Crohn's disease patients; UC = ulcerative colitis patients; TMD = temporomandibular disorders; RAS = recurrent aphthous stomatitis; *N* = number(s).

**Table 3:** PSQI and BDI-II test scores in CD, UC patients, and healthy controls.

	HC (*N* 47)	CD (*N* 28)	UC (*N* 19)	*p* value
PSQI > 5, *N* (%)	8 (17.02%)	12 (41.4%)	6 (31.6%)	0.06
BDI-II				
No depression, *N* (%)	44 (93.7%)	21 (72.5%)	15 (79%)	
Mild, *N* (%)	2 (4.2%)	5 (17.2%)	3 (15.8%)	
Moderate, *N* (%)	1 (2.1%)	3 (10.3%)	1 (5.2%)	0.14
Severe, *N* (%)	0	0	0	

PSQI = Pittsburgh Sleep Quality Index, a PSQI global score > 5 is suggestive of significant sleep disturbance; BDI-II = Beck Depression Inventory Scale II; HC = healthy controls; CD = Crohn's disease patients; UC = ulcerative colitis patients; *N* = number(s).
